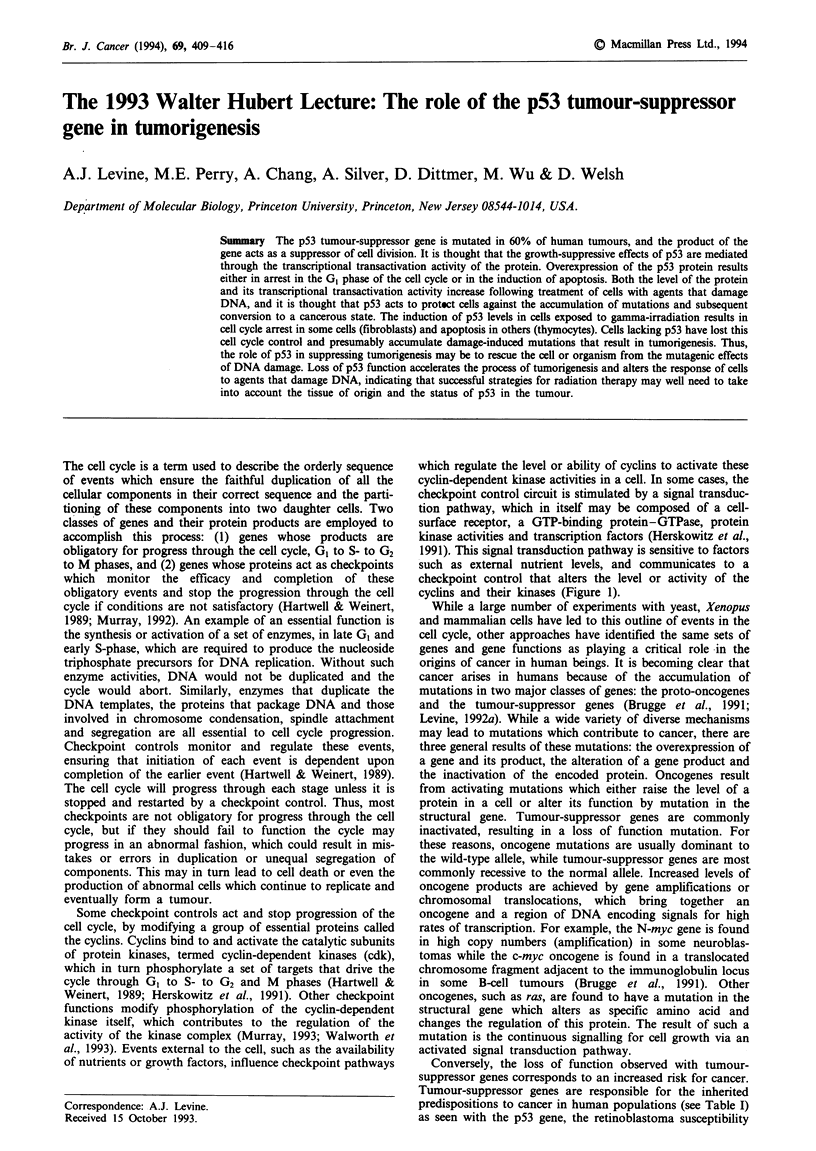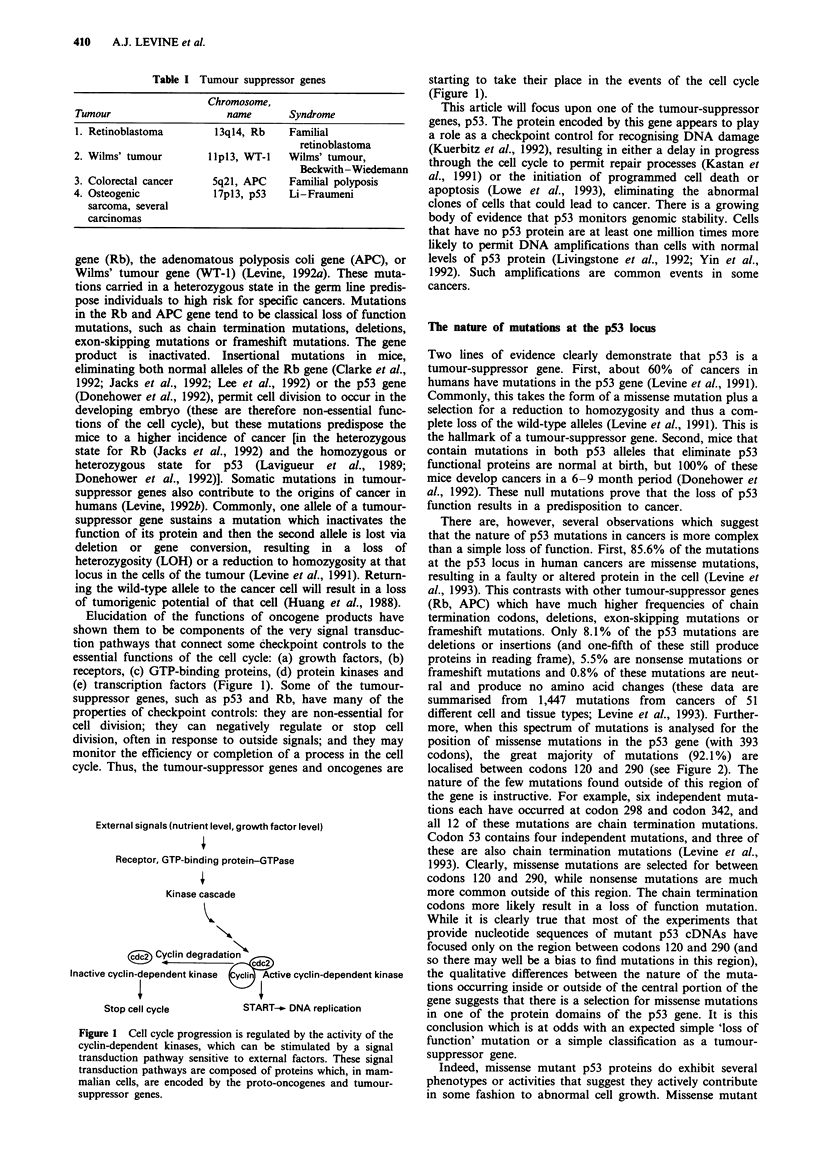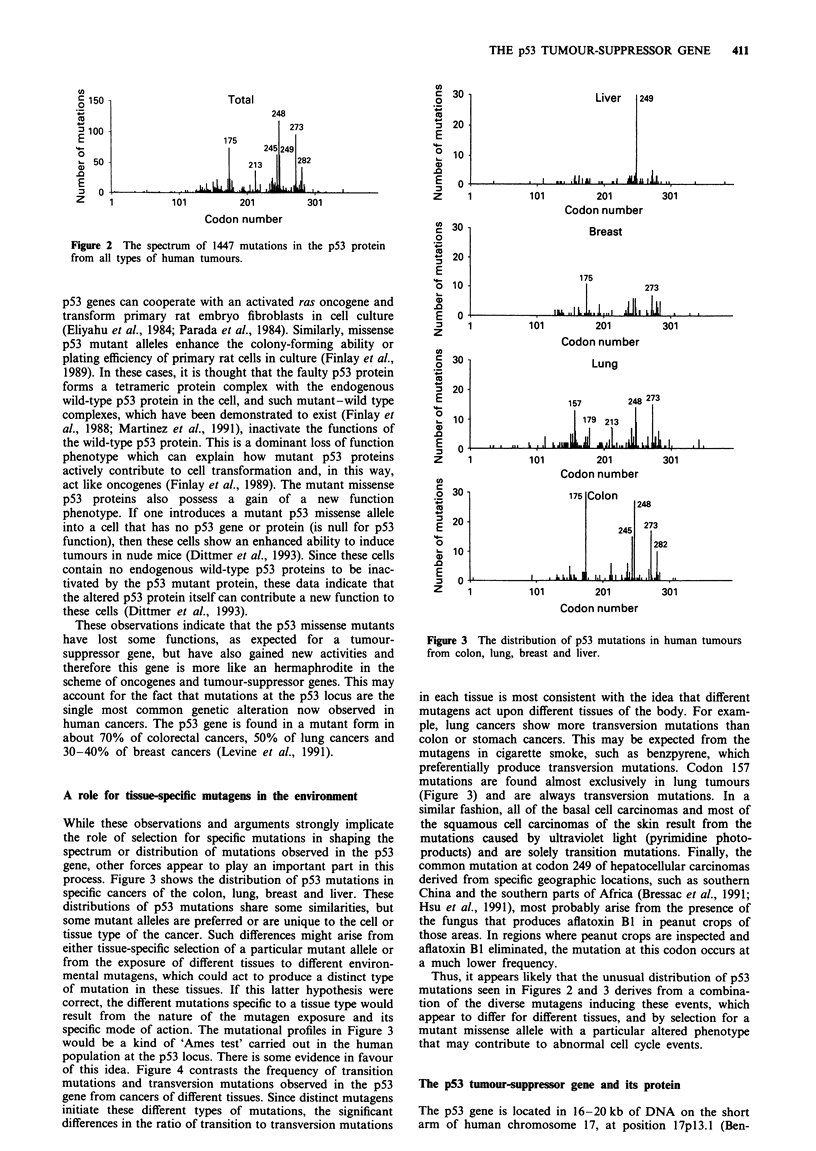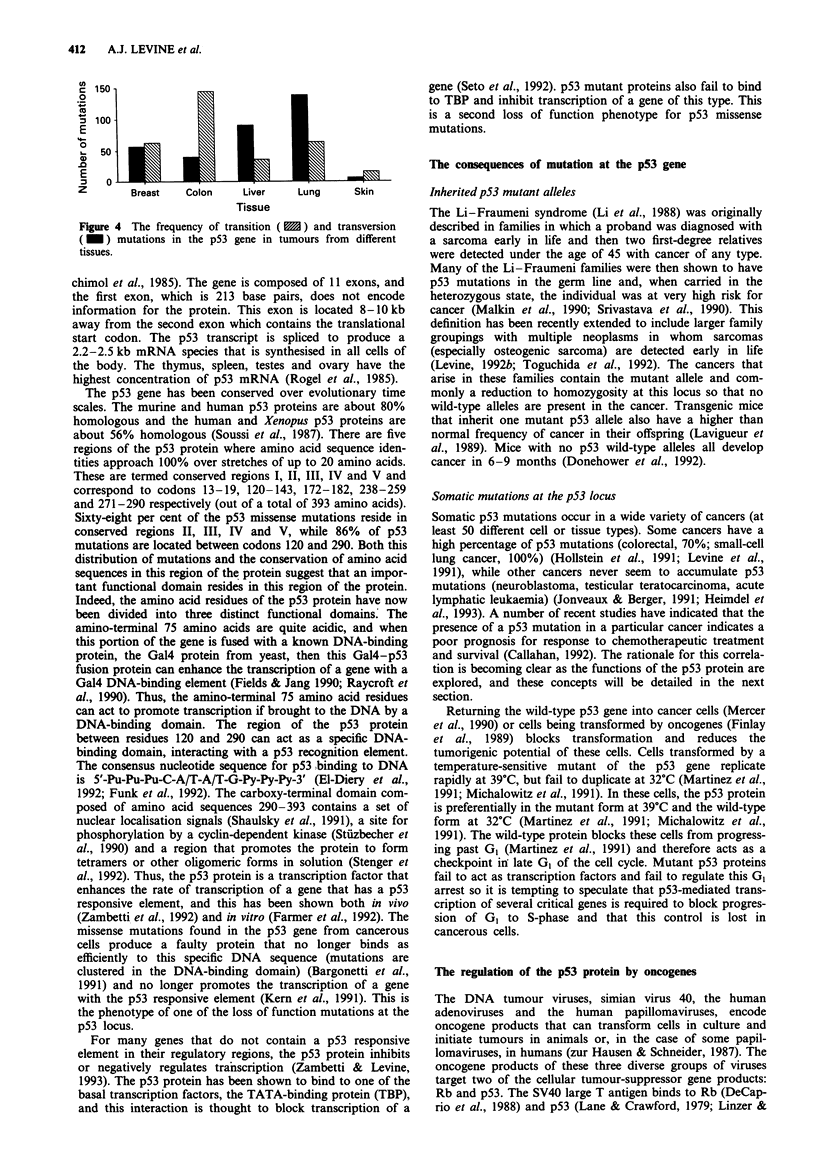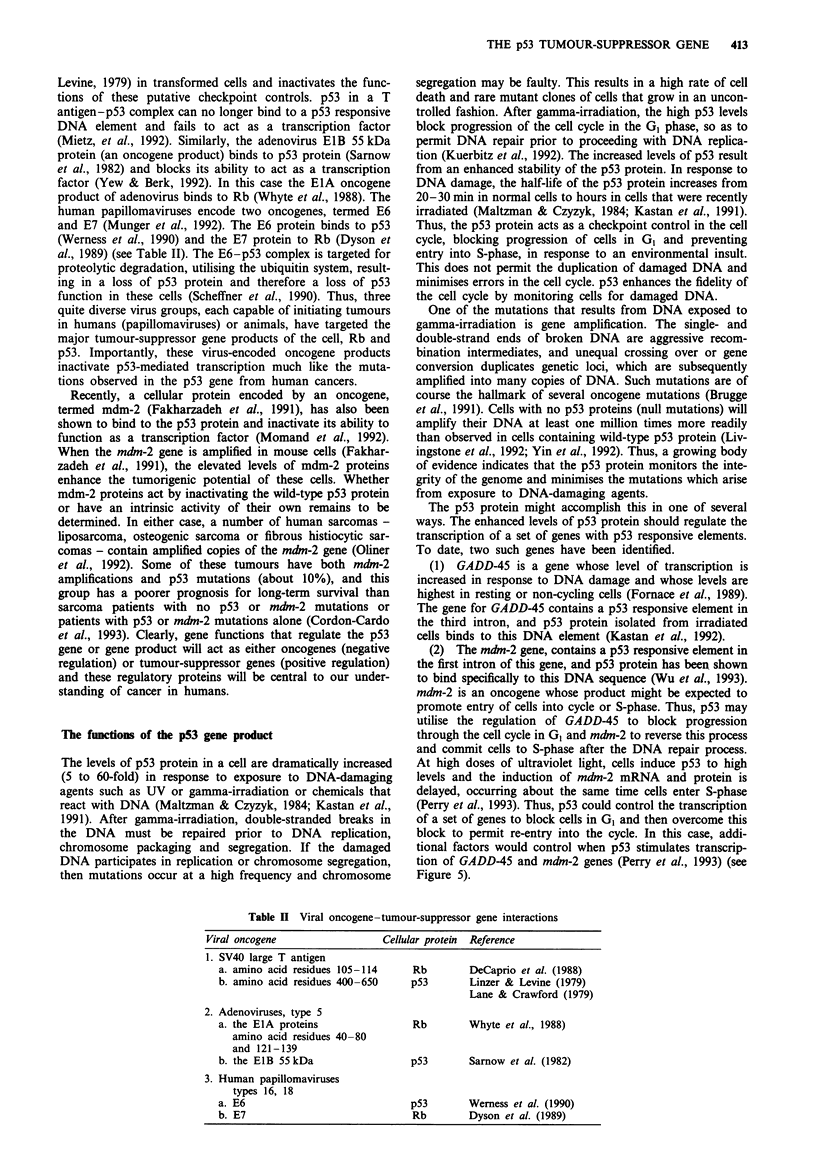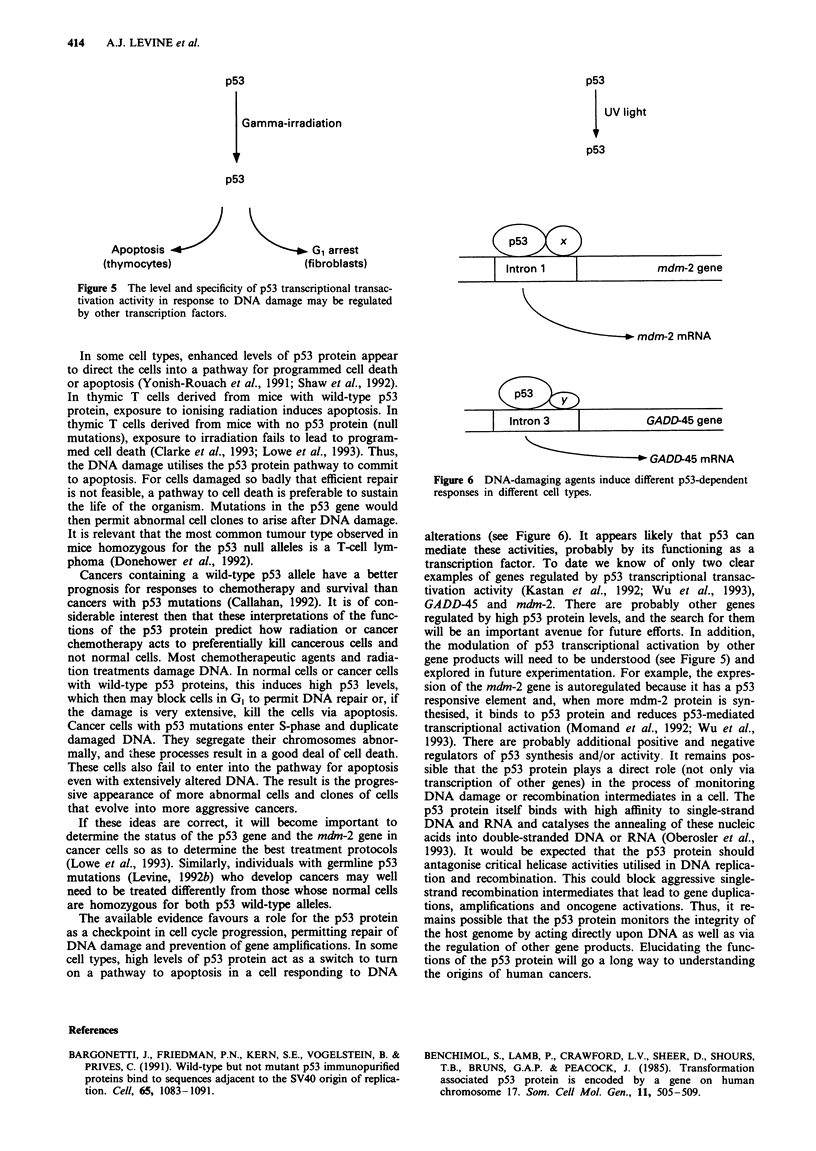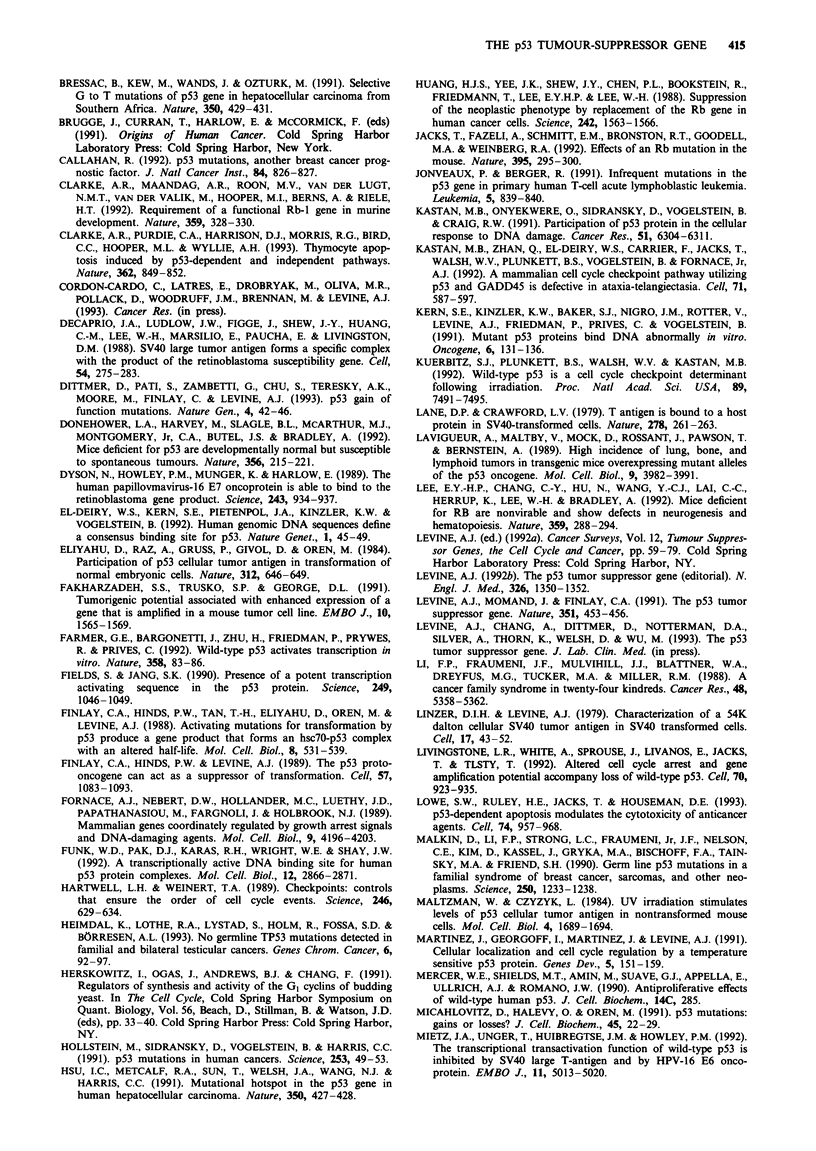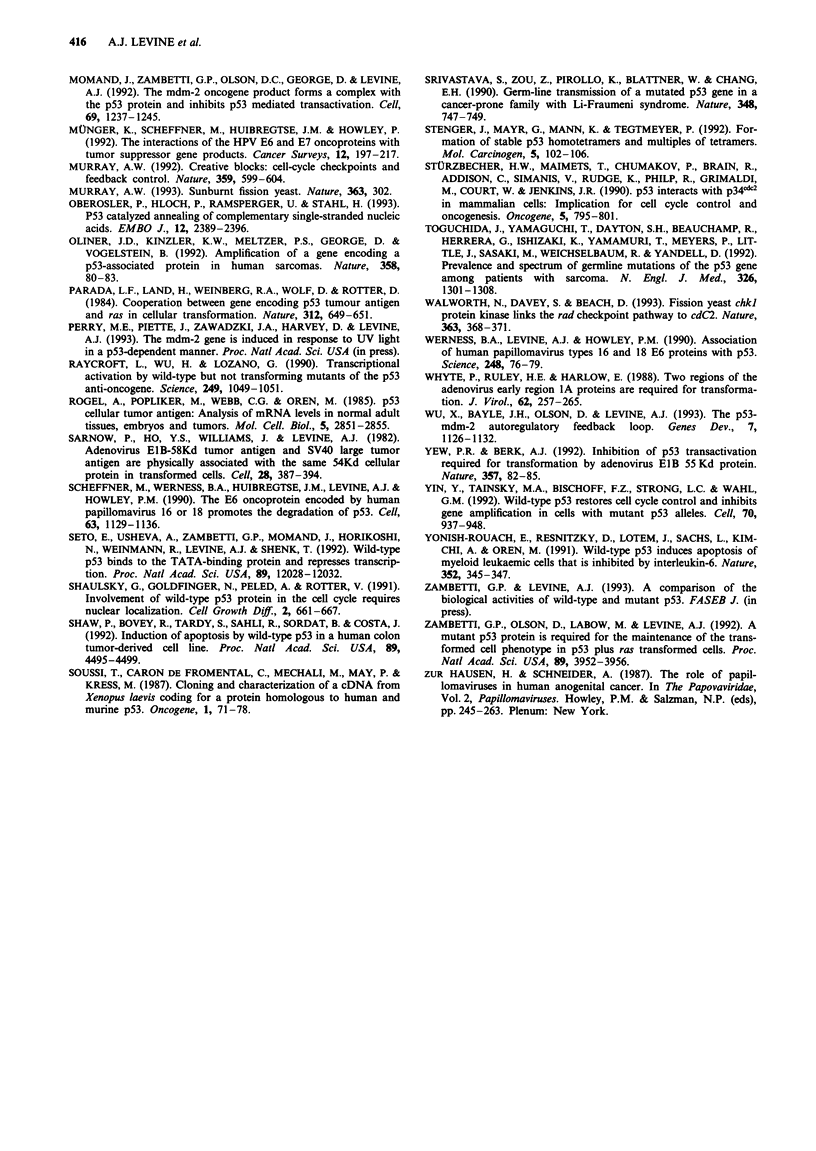# The 1993 Walter Hubert Lecture: the role of the p53 tumour-suppressor gene in tumorigenesis.

**DOI:** 10.1038/bjc.1994.76

**Published:** 1994-03

**Authors:** A. J. Levine, M. E. Perry, A. Chang, A. Silver, D. Dittmer, M. Wu, D. Welsh

**Affiliations:** Department of Molecular Biology, Princeton University, New Jersey 08544-1014.

## Abstract

The p53 tumour-suppressor gene is mutated in 60% of human tumours, and the product of the gene acts as a suppressor of cell division. It is thought that the growth-suppressive effects of p53 are mediated through the transcriptional transactivation activity of the protein. Overexpression of the p53 protein results either in arrest in the G1 phase of the cell cycle or in the induction of apoptosis. Both the level of the protein and its transcriptional transactivation activity increase following treatment of cells with agents that damage DNA, and it is thought that p53 acts to protect cells against the accumulation of mutations and subsequent conversion to a cancerous state. The induction of p53 levels in cells exposed to gamma-irradiation results in cell cycle arrest in some cells (fibroblasts) and apoptosis in others (thymocytes). Cells lacking p53 have lost this cell cycle control and presumably accumulate damage-induced mutations that result in tumorigenesis. Thus, the role of p53 in suppressing tumorigenesis may be to rescue the cell or organism from the mutagenic effects of DNA damage. Loss of p53 function accelerates the process of tumorigenesis and alters the response of cells to agents that damage DNA, indicating that successful strategies for radiation therapy may well need to take into account the tissue of origin and the status of p53 in the tumour.


Br. J. Cancer (1994), 69, 409-416 © Macmillan Press Ltd., 1994

### The 1993 Walter Hubert Lecture: The role of the p53 tumour-suppressor gene in tumorigenesis

A.J. Levine, M.E. Perry, A. Chang, A. Silver, D. Dittmer, M. Wu & D. Welsh

*Department of Molecular Biology, Princeton University, Princeton, New Jersey 08544-1014, USA.*

**Summary** The p53 tumour-suppressor gene is mutated in 60% of human tumours, and the product of the gene acts as a suppressor of cell division. It is thought that the growth-suppressive effects of p53 are mediated through the transcriptional transactivation activity of the protein. Overexpression of the p53 protein results either in arrest in the $G_1$ phase of the cell cycle or in the induction of apoptosis. Both the level of the protein and its transcriptional transactivation activity increase following treatment of cells with agents that damage DNA, and it is thought that p53 acts to protect cells against the accumulation of mutations and subsequent conversion to a cancerous state. The induction of p53 levels in cells exposed to gamma-irradiation results in cell cycle arrest in some cells (fibroblasts) and apoptosis in others (thymocytes). Cells lacking p53 have lost this cell cycle control and presumably accumulate damage-induced mutations that result in tumorigenesis. Thus, the role of p53 in suppressing tumorigenesis may be to rescue the cell or organism from the mutagenic effects of DNA damage. Loss of p53 function accelerates the process of tumorigenesis and alters the response of cells to agents that damage DNA, indicating that successful strategies for radiation therapy may well need to take into account the tissue of origin and the status of p53 in the tumour.

The cell cycle is a term used to describe the orderly sequence of events which ensure the faithful duplication of all the cellular components in their correct sequence and the partitioning of these components into two daughter cells. Two classes of genes and their protein products are employed to accomplish this process: (1) genes whose products are obligatory for progress through the cell cycle, $G_1$ to S- to $G_2$ to M phases, and (2) genes whose proteins act as checkpoints which monitor the efficacy and completion of these obligatory events and stop the progression through the cell cycle if conditions are not satisfactory (Hartwell & Weinert, 1989; Murray, 1992). An example of an essential function is the synthesis or activation of a set of enzymes, in late $G_1$ and early S-phase, which are required to produce the nucleoside triphosphate precursors for DNA replication. Without such enzyme activities, DNA would not be duplicated and the cycle would abort. Similarly, enzymes that duplicate the DNA templates, the proteins that package DNA and those involved in chromosome condensation, spindle attachment and segregation are all essential to cell cycle progression. Checkpoint controls monitor and regulate these events, ensuring that initiation of each event is dependent upon completion of the earlier event (Hartwell & Weinert, 1989). The cell cycle will progress through each stage unless it is stopped and restarted by a checkpoint control. Thus, most checkpoints are not obligatory for progress through the cell cycle, but if they should fail to function the cycle may progress in an abnormal fashion, which could result in mistakes or errors in duplication or unequal segregation of components. This may in turn lead to cell death or even the production of abnormal cells which continue to replicate and eventually form a tumour.

Some checkpoint controls act and stop progression of the cell cycle, by modifying a group of essential proteins called the cyclins. Cyclins bind to and activate the catalytic subunits of protein kinases, termed cyclin-dependent kinases (cdk), which in turn phosphorylate a set of targets that drive the cycle through $G_1$ to S- to $G_2$ and M phases (Hartwell & Weinert, 1989; Herskowitz *et al.*, 1991). Other checkpoint functions modify phosphorylation of the cyclin-dependent kinase itself, which contributes to the regulation of the activity of the kinase complex (Murray, 1993; Walworth *et al.*, 1993). Events external to the cell, such as the availability of nutrients or growth factors, influence checkpoint pathways which regulate the level or ability of cyclins to activate these cyclin-dependent kinase activities in a cell. In some cases, the checkpoint control circuit is stimulated by a signal transduction pathway, which in itself may be composed of a cell-surface receptor, a GTP-binding protein-GTPase, protein kinase activities and transcription factors (Herskowitz *et al.*, 1991). This signal transduction pathway is sensitive to factors such as external nutrient levels, and communicates to a checkpoint control that alters the level or activity of the cyclins and their kinases (Figure 1).

While a large number of experiments with yeast, *Xenopus* and mammalian cells have led to this outline of events in the cell cycle, other approaches have identified the same sets of genes and gene functions as playing a critical role in the origins of cancer in human beings. It is becoming clear that cancer arises in humans because of the accumulation of mutations in two major classes of genes: the proto-oncogenes and the tumour-suppressor genes (Brugge *et al.*, 1991; Levine, 1992*a*). While a wide variety of diverse mechanisms may lead to mutations which contribute to cancer, there are three general results of these mutations: the overexpression of a gene and its product, the alteration of a gene product and the inactivation of the encoded protein. Oncogenes result from activating mutations which either raise the level of a protein in a cell or alter its function by mutation in the structural gene. Tumour-suppressor genes are commonly inactivated, resulting in a loss of function mutation. For these reasons, oncogene mutations are usually dominant to the wild-type allele, while tumour-suppressor genes are most commonly recessive to the normal allele. Increased levels of oncogene products are achieved by gene amplifications or chromosomal translocations, which bring together an oncogene and a region of DNA encoding signals for high rates of transcription. For example, the N-*myc* gene is found in high copy numbers (amplification) in some neuroblastomas while the c-*myc* oncogene is found in a translocated chromosome fragment adjacent to the immunoglobulin locus in some B-cell tumours (Brugge *et al.*, 1991). Other oncogenes, such as *ras*, are found to have a mutation in the structural gene which alters as specific amino acid and changes the regulation of this protein. The result of such a mutation is the continuous signalling for cell growth via an activated signal transduction pathway.

Conversely, the loss of function observed with tumour-suppressor genes corresponds to an increased risk for cancer. Tumour-suppressor genes are responsible for the inherited predispositions to cancer in human populations (see Table I) as seen with the p53 gene, the retinoblastoma susceptibility

Correspondence: A.J. Levine.
Received 15 October 1993.

**Table I** Tumour suppressor genes

| Tumour | Chromosome, name | Syndrome |
|---|---|---|
| 1. Retinoblastoma | 13q14, Rb | Familial retinoblastoma |
| 2. Wilms' tumour | 11p13, WT-1 | Wilms' tumour, Beckwith-Wiedemann |
| 3. Colorectal cancer | 5q21, APC | Familial polyposis |
| 4. Osteogenic sarcoma, several carcinomas | 17p13, p53 | Li-Fraumeni |

gene (Rb), the adenomatous polyposis coli gene (APC), or Wilms' tumour gene (WT-1) (Levine, 1992*a*). These mutations carried in a heterozygous state in the germ line predispose individuals to high risk for specific cancers. Mutations in the Rb and APC gene tend to be classical loss of function mutations, such as chain termination mutations, deletions, exon-skipping mutations or frameshift mutations. The gene product is inactivated. Insertional mutations in mice, eliminating both normal alleles of the Rb gene (Clarke *et al.*, 1992; Jacks *et al.*, 1992; Lee *et al.*, 1992) or the p53 gene (Donehower *et al.*, 1992), permit cell division to occur in the developing embryo (these are therefore non-essential functions of the cell cycle), but these mutations predispose the mice to a higher incidence of cancer [in the heterozygous state for Rb (Jacks *et al.*, 1992) and the homozygous or heterozygous state for p53 (Lavigueur *et al.*, 1989; Donehower *et al.*, 1992)]. Somatic mutations in tumour-suppressor genes also contribute to the origins of cancer in humans (Levine, 1992*b*). Commonly, one allele of a tumour-suppressor gene sustains a mutation which inactivates the function of its protein and then the second allele is lost via deletion or gene conversion, resulting in a loss of heterozygosity (LOH) or a reduction to homozygosity at that locus in the cells of the tumour (Levine *et al.*, 1991). Returning the wild-type allele to the cancer cell will result in a loss of tumorigenic potential of that cell (Huang *et al.*, 1988).

Elucidation of the functions of oncogene products have shown them to be components of the very signal transduction pathways that connect some checkpoint controls to the essential functions of the cell cycle: (a) growth factors, (b) receptors, (c) GTP-binding proteins, (d) protein kinases and (e) transcription factors (Figure 1). Some of the tumour-suppressor genes, such as p53 and Rb, have many of the properties of checkpoint controls: they are non-essential for cell division; they can negatively regulate or stop cell division, often in response to outside signals; and they may monitor the efficiency or completion of a process in the cell cycle. Thus, the tumour-suppressor genes and oncogenes are starting to take their place in the events of the cell cycle (Figure 1).

**Figure 1** Cell cycle progression is regulated by the activity of the cyclin-dependent kinases, which can be stimulated by a signal transduction pathway sensitive to external factors. These signal transduction pathways are composed of proteins which, in mammalian cells, are encoded by the proto-oncogenes and tumour-suppressor genes.

This article will focus upon one of the tumour-suppressor genes, p53. The protein encoded by this gene appears to play a role as a checkpoint control for recognising DNA damage (Kuerbitz *et al.*, 1992), resulting in either a delay in progress through the cell cycle to permit repair processes (Kastan *et al.*, 1991) or the initiation of programmed cell death or apoptosis (Lowe *et al.*, 1993), eliminating the abnormal clones of cells that could lead to cancer. There is a growing body of evidence that p53 monitors genomic stability. Cells that have no p53 protein are at least one million times more likely to permit DNA amplifications than cells with normal levels of p53 protein (Livingstone *et al.*, 1992; Yin *et al.*, 1992). Such amplifications are common events in some cancers.

### The nature of mutations at the p53 locus

Two lines of evidence clearly demonstrate that p53 is a tumour-suppressor gene. First, about 60% of cancers in humans have mutations in the p53 gene (Levine *et al.*, 1991). Commonly, this takes the form of a missense mutation plus a selection for a reduction to homozygosity and thus a complete loss of the wild-type alleles (Levine *et al.*, 1991). This is the hallmark of a tumour-suppressor gene. Second, mice that contain mutations in both p53 alleles that eliminate p53 functional proteins are normal at birth, but 100% of these mice develop cancers in a 6–9 month period (Donehower *et al.*, 1992). These null mutations prove that the loss of p53 function results in a predisposition to cancer.

There are, however, several observations which suggest that the nature of p53 mutations in cancers is more complex than a simple loss of function. First, 85.6% of the mutations at the p53 locus in human cancers are missense mutations, resulting in a faulty or altered protein in the cell (Levine *et al.*, 1993). This contrasts with other tumour-suppressor genes (Rb, APC) which have much higher frequencies of chain termination codons, deletions, exon-skipping mutations or frameshift mutations. Only 8.1% of the p53 mutations are deletions or insertions (and one-fifth of these still produce proteins in reading frame), 5.5% are nonsense mutations or frameshift mutations and 0.8% of these mutations are neutral and produce no amino acid changes (these data are summarised from 1,447 mutations from cancers of 51 different cell and tissue types; Levine *et al.*, 1993). Furthermore, when this spectrum of mutations is analysed for the position of missense mutations in the p53 gene (with 393 codons), the great majority of mutations (92.1%) are localised between codons 120 and 290 (see Figure 2). The nature of the few mutations found outside of this region of the gene is instructive. For example, six independent mutations each have occurred at codon 298 and codon 342, and all 12 of these mutations are chain termination mutations. Codon 53 contains four independent mutations, and three of these are also chain termination mutations (Levine *et al.*, 1993). Clearly, missense mutations are selected for between codons 120 and 290, while nonsense mutations are much more common outside of this region. The chain termination codons more likely result in a loss of function mutation. While it is clearly true that most of the experiments that provide nucleotide sequences of mutant p53 cDNAs have focused only on the region between codons 120 and 290 (and so there may well be a bias to find mutations in this region), the qualitative differences between the nature of the mutations occurring inside or outside of the central portion of the gene suggests that there is a selection for missense mutations in one of the protein domains of the p53 gene. It is this conclusion which is at odds with an expected simple 'loss of function' mutation or a simple classification as a tumour-suppressor gene.

Indeed, missense mutant p53 proteins do exhibit several phenotypes or activities that suggest they actively contribute in some fashion to abnormal cell growth. Missense mutant

**Figure 2** The spectrum of 1447 mutations in the p53 protein from all types of human tumours.

p53 genes can cooperate with an activated *ras* oncogene and transform primary rat embryo fibroblasts in cell culture (Eliyahu *et al.*, 1984; Parada *et al.*, 1984). Similarly, missense p53 mutant alleles enhance the colony-forming ability or plating efficiency of primary rat cells in culture (Finlay *et al.*, 1989). In these cases, it is thought that the faulty p53 protein forms a tetrameric protein complex with the endogenous wild-type p53 protein in the cell, and such mutant–wild type complexes, which have been demonstrated to exist (Finlay *et al.*, 1988; Martinez *et al.*, 1991), inactivate the functions of the wild-type p53 protein. This is a dominant loss of function phenotype which can explain how mutant p53 proteins actively contribute to cell transformation and, in this way, act like oncogenes (Finlay *et al.*, 1989). The mutant missense p53 proteins also possess a gain of a new function phenotype. If one introduces a mutant p53 missense allele into a cell that has no p53 gene or protein (is null for p53 function), then these cells show an enhanced ability to induce tumours in nude mice (Dittmer *et al.*, 1993). Since these cells contain no endogenous wild-type p53 proteins to be inactivated by the p53 mutant protein, these data indicate that the altered p53 protein itself can contribute a new function to these cells (Dittmer *et al.*, 1993).

These observations indicate that the p53 missense mutants have lost some functions, as expected for a tumour-suppressor gene, but have also gained new activities and therefore this gene is more like an hermaphrodite in the scheme of oncogenes and tumour-suppressor genes. This may account for the fact that mutations at the p53 locus are the single most common genetic alteration now observed in human cancers. The p53 gene is found in a mutant form in about 70% of colorectal cancers, 50% of lung cancers and 30–40% of breast cancers (Levine *et al.*, 1991).

### A role for tissue-specific mutagens in the environment

While these observations and arguments strongly implicate the role of selection for specific mutations in shaping the spectrum or distribution of mutations observed in the p53 gene, other forces appear to play an important part in this process. Figure 3 shows the distribution of p53 mutations in specific cancers of the colon, lung, breast and liver. These distributions of p53 mutations share some similarities, but some mutant alleles are preferred or are unique to the cell or tissue type of the cancer. Such differences might arise from either tissue-specific selection of a particular mutant allele or from the exposure of different tissues to different environmental mutagens, which could act to produce a distinct type of mutation in these tissues. If this latter hypothesis were correct, the different mutations specific to a tissue type would result from the nature of the mutagen exposure and its specific mode of action. The mutational profiles in Figure 3 would be a kind of 'Ames test' carried out in the human population at the p53 locus. There is some evidence in favour of this idea. Figure 4 contrasts the frequency of transition mutations and transversion mutations observed in the p53 gene from cancers of different tissues. Since distinct mutagens initiate these different types of mutations, the significant differences in the ratio of transition to transversion mutations

**Figure 3** The distribution of p53 mutations in human tumours from colon, lung, breast and liver.

in each tissue is most consistent with the idea that different mutagens act upon different tissues of the body. For example, lung cancers show more transversion mutations than colon or stomach cancers. This may be expected from the mutagens in cigarette smoke, such as benzpyrene, which preferentially produce transversion mutations. Codon 157 mutations are found almost exclusively in lung tumours (Figure 3) and are always transversion mutations. In a similar fashion, all of the basal cell carcinomas and most of the squamous cell carcinomas of the skin result from the mutations caused by ultraviolet light (pyrimidine photoproducts) and are solely transition mutations. Finally, the common mutation at codon 249 of hepatocellular carcinomas derived from specific geographic locations, such as southern China and the southern parts of Africa (Bressac *et al.*, 1991; Hsu *et al.*, 1991), most probably arise from the presence of the fungus that produces aflatoxin B1 in peanut crops of those areas. In regions where peanut crops are inspected and aflatoxin B1 eliminated, the mutation at this codon occurs at a much lower frequency.

Thus, it appears likely that the unusual distribution of p53 mutations seen in Figures 2 and 3 derives from a combination of the diverse mutagens inducing these events, which appear to differ for different tissues, and by selection for a mutant missense allele with a particular altered phenotype that may contribute to abnormal cell cycle events.

### The p53 tumour-suppressor gene and its protein

The p53 gene is located in 16–20 kb of DNA on the short arm of human chromosome 17, at position 17p13.1 (Ben-

**Figure 4** The frequency of transition (▨) and transversion (■) mutations in the p53 gene in tumours from different tissues.

chimol et al., 1985). The gene is composed of 11 exons, and the first exon, which is 213 base pairs, does not encode information for the protein. This exon is located 8–10 kb away from the second exon which contains the translational start codon. The p53 transcript is spliced to produce a 2.2–2.5 kb mRNA species that is synthesised in all cells of the body. The thymus, spleen, testes and ovary have the highest concentration of p53 mRNA (Rogel et al., 1985).

The p53 gene has been conserved over evolutionary time scales. The murine and human p53 proteins are about 80% homologous and the human and *Xenopus* p53 proteins are about 56% homologous (Soussi et al., 1987). There are five regions of the p53 protein where amino acid sequence identities approach 100% over stretches of up to 20 amino acids. These are termed conserved regions I, II, III, IV and V and correspond to codons 13–19, 120–143, 172–182, 238–259 and 271–290 respectively (out of a total of 393 amino acids). Sixty-eight per cent of the p53 missense mutations reside in conserved regions II, III, IV and V, while 86% of p53 mutations are located between codons 120 and 290. Both this distribution of mutations and the conservation of amino acid sequences in this region of the protein suggest that an important functional domain resides in this region of the protein. Indeed, the amino acid residues of the p53 protein have now been divided into three distinct functional domains. The amino-terminal 75 amino acids are quite acidic, and when this portion of the gene is fused with a known DNA-binding protein, the Gal4 protein from yeast, then this Gal4–p53 fusion protein can enhance the transcription of a gene with a Gal4 DNA-binding element (Fields & Jang 1990; Raycroft et al., 1990). Thus, the amino-terminal 75 amino acid residues can act to promote transcription if brought to the DNA by a DNA-binding domain. The region of the p53 protein between residues 120 and 290 can act as a specific DNA-binding domain, interacting with a p53 recognition element. The consensus nucleotide sequence for p53 binding to DNA is 5′-Pu-Pu-Pu-C-A/T-A/T-G-Py-Py-Py-3′ (El-Diery et al., 1992; Funk et al., 1992). The carboxy-terminal domain composed of amino acid sequences 290–393 contains a set of nuclear localisation signals (Shaulsky et al., 1991), a site for phosphorylation by a cyclin-dependent kinase (Stüzbecher et al., 1990) and a region that promotes the protein to form tetramers or other oligomeric forms in solution (Stenger et al., 1992). Thus, the p53 protein is a transcription factor that enhances the rate of transcription of a gene that has a p53 responsive element, and this has been shown both *in vivo* (Zambetti et al., 1992) and *in vitro* (Farmer et al., 1992). The missense mutations found in the p53 gene from cancerous cells produce a faulty protein that no longer binds as efficiently to this specific DNA sequence (mutations are clustered in the DNA-binding domain) (Bargonetti et al., 1991) and no longer promotes the transcription of a gene with the p53 responsive element (Kern et al., 1991). This is the phenotype of one of the loss of function mutations at the p53 locus.

For many genes that do not contain a p53 responsive element in their regulatory regions, the p53 protein inhibits or negatively regulates transcription (Zambetti & Levine, 1993). The p53 protein has been shown to bind to one of the basal transcription factors, the TATA-binding protein (TBP), and this interaction is thought to block transcription of a gene (Seto et al., 1992). p53 mutant proteins also fail to bind to TBP and inhibit transcription of a gene of this type. This is a second loss of function phenotype for p53 missense mutations.

### The consequences of mutation at the p53 gene

### *Inherited p53 mutant alleles*

The Li–Fraumeni syndrome (Li et al., 1988) was originally described in families in which a proband was diagnosed with a sarcoma early in life and then two first-degree relatives were detected under the age of 45 with cancer of any type. Many of the Li–Fraumeni families were then shown to have p53 mutations in the germ line and, when carried in the heterozygous state, the individual was at very high risk for cancer (Malkin et al., 1990; Srivastava et al., 1990). This definition has been recently extended to include larger family groupings with multiple neoplasms in whom sarcomas (especially osteogenic sarcoma) are detected early in life (Levine, 1992*b*; Toguchida et al., 1992). The cancers that arise in these families contain the mutant allele and commonly a reduction to homozygosity at this locus so that no wild-type alleles are present in the cancer. Transgenic mice that inherit one mutant p53 allele also have a higher than normal frequency of cancer in their offspring (Lavigueur et al., 1989). Mice with no p53 wild-type alleles all develop cancer in 6–9 months (Donehower et al., 1992).

### *Somatic mutations at the p53 locus*

Somatic p53 mutations occur in a wide variety of cancers (at least 50 different cell or tissue types). Some cancers have a high percentage of p53 mutations (colorectal, 70%; small-cell lung cancer, 100%) (Hollstein et al., 1991; Levine et al., 1991), while other cancers never seem to accumulate p53 mutations (neuroblastoma, testicular teratocarcinoma, acute lymphatic leukaemia) (Jonveaux & Berger, 1991; Heimdel et al., 1993). A number of recent studies have indicated that the presence of a p53 mutation in a particular cancer indicates a poor prognosis for response to chemotherapeutic treatment and survival (Callahan, 1992). The rationale for this correlation is becoming clear as the functions of the p53 protein are explored, and these concepts will be detailed in the next section.

Returning the wild-type p53 gene into cancer cells (Mercer et al., 1990) or cells being transformed by oncogenes (Finlay et al., 1989) blocks transformation and reduces the tumorigenic potential of these cells. Cells transformed by a temperature-sensitive mutant of the p53 gene replicate rapidly at 39°C, but fail to duplicate at 32°C (Martinez et al., 1991; Michalowitz et al., 1991). In these cells, the p53 protein is preferentially in the mutant form at 39°C and the wild-type form at 32°C (Martinez et al., 1991; Michalowitz et al., 1991). The wild-type protein blocks these cells from progressing past $G_1$ (Martinez et al., 1991) and therefore acts as a checkpoint in late $G_1$ of the cell cycle. Mutant p53 proteins fail to act as transcription factors and fail to regulate this $G_1$ arrest so it is tempting to speculate that p53-mediated transcription of several critical genes is required to block progression of $G_1$ to S-phase and that this control is lost in cancerous cells.

### The regulation of the p53 protein by oncogenes

The DNA tumour viruses, simian virus 40, the human adenoviruses and the human papillomaviruses, encode oncogene products that can transform cells in culture and initiate tumours in animals or, in the case of some papillomaviruses, in humans (zur Hausen & Schneider, 1987). The oncogene products of these three diverse groups of viruses target two of the cellular tumour-suppressor gene products: Rb and p53. The SV40 large T antigen binds to Rb (DeCaprio et al., 1988) and p53 (Lane & Crawford, 1979; Linzer &

Levine, 1979) in transformed cells and inactivates the functions of these putative checkpoint controls. p53 in a T antigen-p53 complex can no longer bind to a p53 responsive DNA element and fails to act as a transcription factor (Mietz, *et al.*, 1992). Similarly, the adenovirus E1B 55 kDa protein (an oncogene product) binds to p53 protein (Sarnow *et al.*, 1982) and blocks its ability to act as a transcription factor (Yew & Berk, 1992). In this case the E1A oncogene product of adenovirus binds to Rb (Whyte *et al.*, 1988). The human papillomaviruses encode two oncogenes, termed E6 and E7 (Munger *et al.*, 1992). The E6 protein binds to p53 (Werness *et al.*, 1990) and the E7 protein to Rb (Dyson *et al.*, 1989) (see Table II). The E6-p53 complex is targeted for proteolytic degradation, utilising the ubiquitin system, resulting in a loss of p53 protein and therefore a loss of p53 function in these cells (Scheffner *et al.*, 1990). Thus, three quite diverse virus groups, each capable of initiating tumours in humans (papillomaviruses) or animals, have targeted the major tumour-suppressor gene products of the cell, Rb and p53. Importantly, these virus-encoded oncogene products inactivate p53-mediated transcription much like the mutations observed in the p53 gene from human cancers.

Recently, a cellular protein encoded by an oncogene, termed mdm-2 (Fakharzadeh *et al.*, 1991), has also been shown to bind to the p53 protein and inactivate its ability to function as a transcription factor (Momand *et al.*, 1992). When the *mdm*-2 gene is amplified in mouse cells (Fakharzadeh *et al.*, 1991), the elevated levels of mdm-2 proteins enhance the tumorigenic potential of these cells. Whether mdm-2 proteins act by inactivating the wild-type p53 protein or have an intrinsic activity of their own remains to be determined. In either case, a number of human sarcomas - liposarcoma, osteogenic sarcoma or fibrous histiocytic sarcomas - contain amplified copies of the *mdm*-2 gene (Oliner *et al.*, 1992). Some of these tumours have both *mdm*-2 amplifications and p53 mutations (about 10%), and this group has a poorer prognosis for long-term survival than sarcoma patients with no p53 or *mdm*-2 mutations or patients with p53 or *mdm*-2 mutations alone (Cordon-Cardo *et al.*, 1993). Clearly, gene functions that regulate the p53 gene or gene product will act as either oncogenes (negative regulation) or tumour-suppressor genes (positive regulation) and these regulatory proteins will be central to our understanding of cancer in humans.

### The functions of the p53 gene product

The levels of p53 protein in a cell are dramatically increased (5 to 60-fold) in response to exposure to DNA-damaging agents such as UV or gamma-irradiation or chemicals that react with DNA (Maltzman & Czyzyk, 1984; Kastan *et al.*, 1991). After gamma-irradiation, double-stranded breaks in the DNA must be repaired prior to DNA replication, chromosome packaging and segregation. If the damaged DNA participates in replication or chromosome segregation, then mutations occur at a high frequency and chromosome segregation may be faulty. This results in a high rate of cell death and rare mutant clones of cells that grow in an uncontrolled fashion. After gamma-irradiation, the high p53 levels block progression of the cell cycle in the $G_1$ phase, so as to permit DNA repair prior to proceeding with DNA replication (Kuerbitz *et al.*, 1992). The increased levels of p53 result from an enhanced stability of the p53 protein. In response to DNA damage, the half-life of the p53 protein increases from 20-30 min in normal cells to hours in cells that were recently irradiated (Maltzman & Czyzyk, 1984; Kastan *et al.*, 1991). Thus, the p53 protein acts as a checkpoint control in the cell cycle, blocking progression of cells in $G_1$ and preventing entry into S-phase, in response to an environmental insult. This does not permit the duplication of damaged DNA and minimises errors in the cell cycle. p53 enhances the fidelity of the cell cycle by monitoring cells for damaged DNA.

One of the mutations that results from DNA exposed to gamma-irradiation is gene amplification. The single- and double-strand ends of broken DNA are aggressive recombination intermediates, and unequal crossing over or gene conversion duplicates genetic loci, which are subsequently amplified into many copies of DNA. Such mutations are of course the hallmark of several oncogene mutations (Brugge *et al.*, 1991). Cells with no p53 proteins (null mutations) will amplify their DNA at least one million times more readily than observed in cells containing wild-type p53 protein (Livingstone *et al.*, 1992; Yin *et al.*, 1992). Thus, a growing body of evidence indicates that the p53 protein monitors the integrity of the genome and minimises the mutations which arise from exposure to DNA-damaging agents.

The p53 protein might accomplish this in one of several ways. The enhanced levels of p53 protein should regulate the transcription of a set of genes with p53 responsive elements. To date, two such genes have been identified.

(1) *GADD*-45 is a gene whose level of transcription is increased in response to DNA damage and whose levels are highest in resting or non-cycling cells (Fornace *et al.*, 1989). The gene for *GADD*-45 contains a p53 responsive element in the third intron, and p53 protein isolated from irradiated cells binds to this DNA element (Kastan *et al.*, 1992).

(2) The *mdm*-2 gene, contains a p53 responsive element in the first intron of this gene, and p53 protein has been shown to bind specifically to this DNA sequence (Wu *et al.*, 1993). *mdm*-2 is an oncogene whose product might be expected to promote entry of cells into cycle or S-phase. Thus, p53 may utilise the regulation of *GADD*-45 to block progression through the cell cycle in $G_1$ and *mdm*-2 to reverse this process and commit cells to S-phase after the DNA repair process. At high doses of ultraviolet light, cells induce p53 to high levels and the induction of *mdm*-2 mRNA and protein is delayed, occurring about the same time cells enter S-phase (Perry *et al.*, 1993). Thus, p53 could control the transcription of a set of genes to block cells in $G_1$ and then overcome this block to permit re-entry into the cycle. In this case, additional factors would control when p53 stimulates transcription of *GADD*-45 and *mdm*-2 genes (Perry *et al.*, 1993) (see Figure 5).

**Table II** Viral oncogene-tumour-suppressor gene interactions

| *Viral oncogene* | *Cellular protein* | *Reference* |
|---|---|---|
| 1. SV40 large T antigen | | |
| a. amino acid residues 105-114 | Rb | DeCaprio *et al.* (1988) |
| b. amino acid residues 400-650 | p53 | Linzer & Levine (1979) |
| | | Lane & Crawford (1979) |
| 2. Adenoviruses, type 5 | | |
| a. the E1A proteins | Rb | Whyte *et al.*, 1988) |
| amino acid residues 40-80 | | |
| and 121-139 | | |
| b. the E1B 55 kDa | p53 | Sarnow *et al.* (1982) |
| 3. Human papillomaviruses | | |
| types 16, 18 | | |
| a. E6 | p53 | Werness *et al.* (1990) |
| b. E7 | Rb | Dyson *et al.* (1989) |

**Figure 5** The level and specificity of p53 transcriptional transactivation activity in response to DNA damage may be regulated by other transcription factors.

**Figure 6** DNA-damaging agents induce different p53-dependent responses in different cell types.

In some cell types, enhanced levels of p53 protein appear to direct the cells into a pathway for programmed cell death or apoptosis (Yonish-Rouach *et al.*, 1991; Shaw *et al.*, 1992). In thymic T cells derived from mice with wild-type p53 protein, exposure to ionising radiation induces apoptosis. In thymic T cells derived from mice with no p53 protein (null mutations), exposure to irradiation fails to lead to programmed cell death (Clarke *et al.*, 1993; Lowe *et al.*, 1993). Thus, the DNA damage utilises the p53 protein pathway to commit to apoptosis. For cells damaged so badly that efficient repair is not feasible, a pathway to cell death is preferable to sustain the life of the organism. Mutations in the p53 gene would then permit abnormal cell clones to arise after DNA damage. It is relevant that the most common tumour type observed in mice homozygous for the p53 null alleles is a T-cell lymphoma (Donehower *et al.*, 1992).

Cancers containing a wild-type p53 allele have a better prognosis for responses to chemotherapy and survival than cancers with p53 mutations (Callahan, 1992). It is of considerable interest then that these interpretations of the functions of the p53 protein predict how radiation or cancer chemotherapy acts to preferentially kill cancerous cells and not normal cells. Most chemotherapeutic agents and radiation treatments damage DNA. In normal cells or cancer cells with wild-type p53 proteins, this induces high p53 levels, which then may block cells in $G_1$ to permit DNA repair or, if the damage is very extensive, kill the cells via apoptosis. Cancer cells with p53 mutations enter S-phase and duplicate damaged DNA. They segregate their chromosomes abnormally, and these processes result in a good deal of cell death. These cells also fail to enter into the pathway for apoptosis even with extensively altered DNA. The result is the progressive appearance of more abnormal cells and clones of cells that evolve into more aggressive cancers.

If these ideas are correct, it will become important to determine the status of the p53 gene and the *mdm*-2 gene in cancer cells so as to determine the best treatment protocols (Lowe *et al.*, 1993). Similarly, individuals with germline p53 mutations (Levine, 1992*b*) who develop cancers may well need to be treated differently from those whose normal cells are homozygous for both p53 wild-type alleles.

The available evidence favours a role for the p53 protein as a checkpoint in cell cycle progression, permitting repair of DNA damage and prevention of gene amplifications. In some cell types, high levels of p53 protein act as a switch to turn on a pathway to apoptosis in a cell responding to DNA alterations (see Figure 6). It appears likely that p53 can mediate these activities, probably by its functioning as a transcription factor. To date we know of only two clear examples of genes regulated by p53 transcriptional transactivation activity (Kastan *et al.*, 1992; Wu *et al.*, 1993), *GADD*-45 and *mdm*-2. There are probably other genes regulated by high p53 protein levels, and the search for them will be an important avenue for future efforts. In addition, the modulation of p53 transcriptional activation by other gene products will need to be understood (see Figure 5) and explored in future experimentation. For example, the expression of the *mdm*-2 gene is autoregulated because it has a p53 responsive element and, when more mdm-2 protein is synthesised, it binds to p53 protein and reduces p53-mediated transcriptional activation (Momand *et al.*, 1992; Wu *et al.*, 1993). There are probably additional positive and negative regulators of p53 synthesis and/or activity. It remains possible that the p53 protein plays a direct role (not only via transcription of other genes) in the process of monitoring DNA damage or recombination intermediates in a cell. The p53 protein itself binds with high affinity to single-strand DNA and RNA and catalyses the annealing of these nucleic acids into double-stranded DNA or RNA (Oberosler *et al.*, 1993). It would be expected that the p53 protein should antagonise critical helicase activities utilised in DNA replication and recombination. This could block aggressive single-strand recombination intermediates that lead to gene duplications, amplifications and oncogene activations. Thus, it remains possible that the p53 protein monitors the integrity of the host genome by acting directly upon DNA as well as via the regulation of other gene products. Elucidating the functions of the p53 protein will go a long way to understanding the origins of human cancers.

### References

BARGONETTI, J., FRIEDMAN, P.N., KERN, S.E., VOGELSTEIN, B. & PRIVES, C. (1991). Wild-type but not mutant p53 immunopurified proteins bind to sequences adjacent to the SV40 origin of replication. *Cell*, **65**, 1083-1091.

BENCHIMOL, S., LAMB, P., CRAWFORD, L.V., SHEER, D., SHOURS, T.B., BRUNS, G.A.P. & PEACOCK, J. (1985). Transformation associated p53 protein is encoded by a gene on human chromosome 17. *Som. Cell Mol. Gen.*, **11**, 505-509.

BRESSAC, B., KEW, M., WANDS, J. & OZTURK, M. (1991). Selective G to T mutations of p53 gene in hepatocellular carcinoma from Southern Africa. *Nature*, **350**, 429–431.

BRUGGE, J., CURRAN, T., HARLOW, E. & MCCORMICK, F. (eds) (1991). *Origins of Human Cancer*. Cold Spring Harbor Laboratory Press: Cold Spring Harbor, New York.

CALLAHAN, R. (1992). p53 mutations, another breast cancer prognostic factor. *J. Natl Cancer Inst.*, **84**, 826–827.

CLARKE, A.R., MAANDAG, A.R., ROON, M.V., VAN DER LUGT, N.M.T., VAN DER VALIK, M., HOOPER, M.I., BERNS, A. & RIELE, H.T. (1992). Requirement of a functional Rb-1 gene in murine development. *Nature*, **359**, 328–330.

CLARKE, A.R., PURDIE, C.A., HARRISON, D.J., MORRIS, R.G., BIRD, C.C., HOOPER, M.L. & WYLLIE, A.H. (1993). Thymocyte apoptosis induced by p53-dependent and independent pathways. *Nature*, **362**, 849–852.

CORDON-CARDO, C., LATRES, E., DROBRYAK, M., OLIVA, M.R., POLLACK, D., WOODRUFF, J.M., BRENNAN, M. & LEVINE, A.J. (1993). *Cancer Res.* (in press).

DECAPRIO, J.A., LUDLOW, J.W., FIGGE, J., SHEW, J.-Y., HUANG, C.-M., LEE, W.-H., MARSILIO, E., PAUCHA, E. & LIVINGSTON, D.M. (1988). SV40 large tumor antigen forms a specific complex with the product of the retinoblastoma susceptibility gene. *Cell*, **54**, 275–283.

DITTMER, D., PATI, S., ZAMBETTI, G., CHU, S., TERESKY, A.K., MOORE, M., FINLAY, C. & LEVINE, A.J. (1993). p53 gain of function mutations. *Nature Gen.*, **4**, 42–46.

DONEHOWER, L.A., HARVEY, M., SLAGLE, B.L., MCARTHUR, M.J., MONTGOMERY, Jr, C.A., BUTEL, J.S. & BRADLEY, A. (1992). Mice deficient for p53 are developmentally normal but susceptible to spontaneous tumours. *Nature*, **356**, 215–221.

DYSON, N., HOWLEY, P.M., MUNGER, K. & HARLOW, E. (1989). The human papillovmavirus-16 E7 oncoprotein is able to bind to the retinoblastoma gene product. *Science*, **243**, 934–937.

EL-DEIRY, W.S., KERN, S.E., PIETENPOL, J.A., KINZLER, K.W. & VOGELSTEIN, B. (1992). Human genomic DNA sequences define a consensus binding site for p53. *Nature Genet.*, **1**, 45–49.

ELIYAHU, D., RAZ, A., GRUSS, P., GIVOL, D. & OREN, M. (1984). Participation of p53 cellular tumor antigen in transformation of normal embryonic cells. *Nature*, **312**, 646–649.

FAKHARZADEH, S.S., TRUSKO, S.P. & GEORGE, D.L. (1991). Tumorigenic potential associated with enhanced expression of a gene that is amplified in a mouse tumor cell line. *EMBO J.*, **10**, 1565–1569.

FARMER, G.E., BARGONETTI, J., ZHU, H., FRIEDMAN, P., PRYWES, R. & PRIVES, C. (1992). Wild-type p53 activates transcription *in vitro*. *Nature*, **358**, 83–86.

FIELDS, S. & JANG, S.K. (1990). Presence of a potent transcription activating sequence in the p53 protein. *Science*, **249**, 1046–1049.

FINLAY, C.A., HINDS, P.W., TAN, T.-H., ELIYAHU, D., OREN, M. & LEVINE, A.J. (1988). Activating mutations for transformation by p53 produce a gene product that forms an hsc70-p53 complex with an altered half-life. *Mol. Cell. Biol.*, **8**, 531–539.

FINLAY, C.A., HINDS, P.W. & LEVINE, A.J. (1989). The p53 proto-oncogene can act as a suppressor of transformation. *Cell*, **57**, 1083–1093.

FORNACE, A.J., NEBERT, D.W., HOLLANDER, M.C., LUETHY, J.D., PAPATHANASIOU, M., FARGNOLI, J. & HOLBROOK, N.J. (1989). Mammalian genes coordinately regulated by growth arrest signals and DNA-damaging agents. *Mol. Cell. Biol.*, **9**, 4196–4203.

FUNK, W.D., PAK, D.J., KARAS, R.H., WRIGHT, W.E. & SHAY, J.W. (1992). A transcriptionally active DNA binding site for human p53 protein complexes. *Mol. Cell. Biol.*, **12**, 2866–2871.

HARTWELL, L.H. & WEINERT, T.A. (1989). Checkpoints: controls that ensure the order of cell cycle events. *Science*, **246**, 629–634.

HEIMDAL, K., LOTHE, R.A., LYSTAD, S., HOLM, R., FOSSA, S.D. & BÖRRESEN, A.L. (1993). No germline TP53 mutations detected in familial and bilateral testicular cancers. *Genes Chrom. Cancer*, **6**, 92–97.

HERSKOWITZ, I., OGAS, J., ANDREWS, B.J. & CHANG, F. (1991). Regulators of synthesis and activity of the $G_1$ cyclins of budding yeast. In *The Cell Cycle*, Cold Spring Harbor Symposium on Quant. Biology, Vol. 56, Beach, D., Stillman, B. & Watson, J.D. (eds), pp. 33–40. Cold Spring Harbor Press: Cold Spring Harbor, NY.

HOLLSTEIN, M., SIDRANSKY, D., VOGELSTEIN, B. & HARRIS, C.C. (1991). p53 mutations in human cancers. *Science*, **253**, 49–53.

HSU, I.C., METCALF, R.A., SUN, T., WELSH, J.A., WANG, N.J. & HARRIS, C.C. (1991). Mutational hotspot in the p53 gene in human hepatocellular carcinoma. *Nature*, **350**, 427–428.

HUANG, H.J.S., YEE, J.K., SHEW, J.Y., CHEN, P.L., BOOKSTEIN, R., FRIEDMANN, T., LEE, E.Y.H.P. & LEE, W.-H. (1988). Suppression of the neoplastic phenotype by replacement of the Rb gene in human cancer cells. *Science*, **242**, 1563–1566.

JACKS, T., FAZELI, A., SCHMITT, E.M., BRONSTON, R.T., GOODELL, M.A. & WEINBERG, R.A. (1992). Effects of an Rb mutation in the mouse. *Nature*, **395**, 295–300.

JONVEAUX, P. & BERGER, R. (1991). Infrequent mutations in the p53 gene in primary human T-cell acute lymphoblastic leukemia. *Leukemia*, **5**, 839–840.

KASTAN, M.B., ONYEKWERE, O., SIDRANSKY, D., VOGELSTEIN, B. & CRAIG, R.W. (1991). Participation of p53 protein in the cellular response to DNA damage. *Cancer Res.*, **51**, 6304–6311.

KASTAN, M.B., ZHAN, Q., EL-DEIRY, W.S., CARRIER, F., JACKS, T., WALSH, W.V., PLUNKETT, B.S., VOGELSTEIN, B. & FORNACE, Jr, A.J. (1992). A mammalian cell cycle checkpoint pathway utilizing p53 and GADD45 is defective in ataxia-telangiectasia. *Cell*, **71**, 587–597.

KERN, S.E., KINZLER, K.W., BAKER, S.J., NIGRO, J.M., ROTTER, V., LEVINE, A.J., FRIEDMAN, P., PRIVES, C. & VOGELSTEIN, B. (1991). Mutant p53 proteins bind DNA abnormally *in vitro*. *Oncogene*, **6**, 131–136.

KUERBITZ, S.J., PLUNKETT, B.S., WALSH, W.V. & KASTAN, M.B. (1992). Wild-type p53 is a cell cycle checkpoint determinant following irradiation. *Proc. Natl Acad. Sci. USA*, **89**, 7491–7495.

LANE, D.P. & CRAWFORD, L.V. (1979). T antigen is bound to a host protein in SV40-transformed cells. *Nature*, **278**, 261–263.

LAVIGUEUR, A., MALTBY, V., MOCK, D., ROSSANT, J., PAWSON, T. & BERNSTEIN, A. (1989). High incidence of lung, bone, and lymphoid tumors in transgenic mice overexpressing mutant alleles of the p53 oncogene. *Mol. Cell. Biol.*, **9**, 3982–3991.

LEE, E.Y.-H.P., CHANG, C.-Y., HU, N., WANG, Y.-C.J., LAI, C.-C., HERRUP, K., LEE, W.-H. & BRADLEY, A. (1992). Mice deficient for RB are nonvirable and show defects in neurogenesis and hematopoiesis. *Nature*, **359**, 288–294.

LEVINE, A.J. (ed.) (1992*a*). *Cancer Surveys*, Vol. 12, *Tumour Suppressor Genes, the Cell Cycle and Cancer*, pp. 59–79. Cold Spring Harbor Laboratory Press: Cold Spring Harbor, NY.

LEVINE, A.J. (1992*b*). The p53 tumor suppressor gene (editorial). *N. Engl. J. Med.*, **326**, 1350–1352.

LEVINE, A.J., MOMAND, J. & FINLAY, C.A. (1991). The p53 tumor suppressor gene. *Nature*, **351**, 453–456.

LEVINE, A.J., CHANG, A., DITTMER, D., NOTTERMAN, D.A., SILVER, A., THORN, K., WELSH, D. & WU, M. (1993). The p53 tumor suppressor gene. *J. Lab. Clin. Med.* (in press).

LI, F.P., FRAUMENI, J.F., MULVIHILL, J.J., BLATTNER, W.A., DREYFUS, M.G., TUCKER, M.A. & MILLER, R.M. (1988). A cancer family syndrome in twenty-four kindreds. *Cancer Res.*, **48**, 5358–5362.

LINZER, D.I.H. & LEVINE, A.J. (1979). Characterization of a 54K dalton cellular SV40 tumor antigen in SV40 transformed cells. *Cell*, **17**, 43–52.

LIVINGSTONE, L.R., WHITE, A., SPROUSE, J., LIVANOS, E., JACKS, T. & TLSTY, T. (1992). Altered cell cycle arrest and gene amplification potential accompany loss of wild-type p53. *Cell*, **70**, 923–935.

LOWE, S.W., RULEY, H.E., JACKS, T. & HOUSEMAN, D.E. (1993). p53-dependent apoptosis modulates the cytotoxicity of anticancer agents. *Cell*, **74**, 957–968.

MALKIN, D., LI, F.P., STRONG, L.C., FRAUMENI, Jr, J.F., NELSON, C.E., KIM, D., KASSEL, J., GRYKA, M.A., BISCHOFF, F.A., TAINSKY, M.A. & FRIEND, S.H. (1990). Germ line p53 mutations in a familial syndrome of breast cancer, sarcomas, and other neoplasms. *Science*, **250**, 1233–1238.

MALTZMAN, W. & CZYZYK, L. (1984). UV irradiation stimulates levels of p53 cellular tumor antigen in nontransformed mouse cells. *Mol. Cell. Biol.* **4**, 1689–1694.

MARTINEZ, J., GEORGOFF, I., MARTINEZ, J. & LEVINE, A.J. (1991). Cellular localization and cell cycle regulation by a temperature sensitive p53 protein. *Genes Dev.*, **5**, 151–159.

MERCER, W.E., SHIELDS, M.T., AMIN, M., SUAVE, G.J., APPELLA, E., ULLRICH, A.J. & ROMANO, J.W. (1990). Antiproliferative effects of wild-type human p53. *J. Cell. Biochem.*, **14C**, 285.

MICAHLOVITZ, D., HALEVY, O. & OREN, M. (1991). p53 mutations: gains or losses? *J. Cell. Biochem.*, **45**, 22–29.

MIETZ, J.A., UNGER, T., HUIBREGTSE, J.M. & HOWLEY, P.M. (1992). The transcriptional transactivation function of wild-type p53 is inhibited by SV40 large T-antigen and by HPV-16 E6 oncoprotein. *EMBO J.*, **11**, 5013–5020.

MOMAND, J., ZAMBETTI, G.P., OLSON, D.C., GEORGE, D. & LEVINE, A.J. (1992). The mdm-2 oncogene product forms a complex with the p53 protein and inhibits p53 mediated transactivation. *Cell*, **69**, 1237–1245.

MÜNGER, K., SCHEFFNER, M., HUIBREGTSE, J.M. & HOWLEY, P. (1992). The interactions of the HPV E6 and E7 oncoproteins with tumor suppressor gene products. *Cancer Surveys*, **12**, 197–217.

MURRAY, A.W. (1992). Creative blocks: cell-cycle checkpoints and feedback control. *Nature*, **359**, 599–604.

MURRAY, A.W. (1993). Sunburnt fission yeast. *Nature*, **363**, 302.

OBEROSLER, P., HLOCH, P., RAMSPERGER, U. & STAHL, H. (1993). P53 catalyzed annealing of complementary single-stranded nucleic acids. *EMBO J.*, **12**, 2389–2396.

OLINER, J.D., KINZLER, K.W., MELTZER, P.S., GEORGE, D. & VOGELSTEIN, B. (1992). Amplification of a gene encoding a p53-associated protein in human sarcomas. *Nature*, **358**, 80–83.

PARADA, L.F., LAND, H., WEINBERG, R.A., WOLF, D. & ROTTER, D. (1984). Cooperation between gene encoding p53 tumour antigen and *ras* in cellular transformation. *Nature*, **312**, 649–651.

PERRY, M.E., PIETTE, J., ZAWADZKI, J.A., HARVEY, D. & LEVINE, A.J. (1993). The mdm-2 gene is induced in response to UV light in a p53-dependent manner. *Proc. Natl Acad. Sci. USA* (in press).

RAYCROFT, L., WU, H. & LOZANO, G. (1990). Transcriptional activation by wild-type but not transforming mutants of the p53 anti-oncogene. *Science*, **249**, 1049–1051.

ROGEL, A., POPLIKER, M., WEBB, C.G. & OREN, M. (1985). p53 cellular tumor antigen: Analysis of mRNA levels in normal adult tissues, embryos and tumors. *Mol. Cell. Biol.*, **5**, 2851–2855.

SARNOW, P., HO, Y.S., WILLIAMS, J. & LEVINE, A.J. (1982). Adenovirus E1B-58Kd tumor antigen and SV40 large tumor antigen are physically associated with the same 54Kd cellular protein in transformed cells. *Cell*, **28**, 387–394.

SCHEFFNER, M., WERNESS, B.A., HUIBREGTSE, J.M., LEVINE, A.J. & HOWLEY, P.M. (1990). The E6 oncoprotein encoded by human papillomavirus 16 or 18 promotes the degradation of p53. *Cell*, **63**, 1129–1136.

SETO, E., USHEVA, A., ZAMBETTI, G.P., MOMAND, J., HORIKOSHI, N., WEINMANN, R., LEVINE, A.J. & SHENK, T. (1992). Wild-type p53 binds to the TATA-binding protein and represses transcription. *Proc. Natl Acad. Sci. USA*, **89**, 12028–12032.

SHAULSKY, G., GOLDFINGER, N., PELED, A. & ROTTER, V. (1991). Involvement of wild-type p53 protein in the cell cycle requires nuclear localization. *Cell Growth Diff.*, **2**, 661–667.

SHAW, P., BOVEY, R., TARDY, S., SAHLI, R., SORDAT, B. & COSTA, J. (1992). Induction of apoptosis by wild-type p53 in a human colon tumor-derived cell line. *Proc. Natl Acad. Sci. USA*, **89**, 4495–4499.

SOUSSI, T., CARON DE FROMENTAL, C., MECHALI, M., MAY, P. & KRESS, M. (1987). Cloning and characterization of a cDNA from *Xenopus laevis* coding for a protein homologous to human and murine p53. *Oncogene*, **1**, 71–78.

SRIVASTAVA, S., ZOU, Z., PIROLLO, K., BLATTNER, W. & CHANG, E.H. (1990). Germ-line transmission of a mutated p53 gene in a cancer-prone family with Li-Fraumeni syndrome. *Nature*, **348**, 747–749.

STENGER, J., MAYR, G., MANN, K. & TEGTMEYER, P. (1992). Formation of stable p53 homotetramers and multiples of tetramers. *Mol. Carcinogen*, **5**, 102–106.

STÜRZBECHER, H.W., MAIMETS, T., CHUMAKOV, P., BRAIN, R., ADDISON, C., SIMANIS, V., RUDGE, K., PHILP, R., GRIMALDI, M., COURT, W. & JENKINS, J.R. (1990). p53 interacts with $p34^{cdc2}$ in mammalian cells: Implication for cell cycle control and oncogenesis. *Oncogene*, **5**, 795–801.

TOGUCHIDA, J., YAMAGUCHI, T., DAYTON, S.H., BEAUCHAMP, R., HERRERA, G., ISHIZAKI, K., YAMAMURI, T., MEYERS, P., LITTLE, J., SASAKI, M., WEICHSELBAUM, R. & YANDELL, D. (1992). Prevalence and spectrum of germline mutations of the p53 gene among patients with sarcoma. *N. Engl. J. Med.*, **326**, 1301–1308.

WALWORTH, N., DAVEY, S. & BEACH, D. (1993). Fission yeast *chk1* protein kinase links the *rad* checkpoint pathway to *cdC2*. *Nature*, **363**, 368–371.

WERNESS, B.A., LEVINE, A.J. & HOWLEY, P.M. (1990). Association of human papillomavirus types 16 and 18 E6 proteins with p53. *Science*, **248**, 76–79.

WHYTE, P., RULEY, H.E. & HARLOW, E. (1988). Two regions of the adenovirus early region 1A proteins are required for transformation. *J. Virol.*, **62**, 257–265.

WU, X., BAYLE, J.H., OLSON, D. & LEVINE, A.J. (1993). The p53-mdm-2 autoregulatory feedback loop. *Genes Dev.*, **7**, 1126–1132.

YEW, P.R. & BERK, A.J. (1992). Inhibition of p53 transactivation required for transformation by adenovirus E1B 55 Kd protein. *Nature*, **357**, 82–85.

YIN, Y., TAINSKY, M.A., BISCHOFF, F.Z., STRONG, L.C. & WAHL, G.M. (1992). Wild-type p53 restores cell cycle control and inhibits gene amplification in cells with mutant p53 alleles. *Cell*, **70**, 937–948.

YONISH-ROUACH, E., RESNITZKY, D., LOTEM, J., SACHS, L., KIMCHI, A. & OREN, M. (1991). Wild-type p53 induces apoptosis of myeloid leukaemic cells that is inhibited by interleukin-6. *Nature*, **352**, 345–347.

ZAMBETTI, G.P. & LEVINE, A.J. (1993). A comparison of the biological activities of wild-type and mutant p53. *FASEB J.* (in press).

ZAMBETTI, G.P., OLSON, D., LABOW, M. & LEVINE, A.J. (1992). A mutant p53 protein is required for the maintenance of the transformed cell phenotype in p53 plus *ras* transformed cells. *Proc. Natl Acad. Sci. USA*, **89**, 3952–3956.

ZUR HAUSEN, H. & SCHNEIDER, A. (1987). The role of papillomaviruses in human anogenital cancer. In *The Papovaviridae*, Vol. 2, *Papillomaviruses*. Howley, P.M. & Salzman, N.P. (eds), pp. 245–263. Plenum: New York.